# Supplementary material for: Role of desolvation on biomolecular liquid–liquid phase separation
Source: eLife. 2026 Oct 9;15:RP111124. doi: 10.7554/eLife.111124 (PMC13652542; doi:10.7554/eLife.111124)
Supplement: Peer review history

# Editor report: eLife Assessment

**DOI:** 10.7554/eLife.111124.3.sa0

**Authors:** Bin Zhang

**Affiliations:** Massachusetts Institute of Technology United States

**Keywords:** Convincing, Important

This article presents an **important** and timely contribution by incorporating desolvation barriers into coarse-grained models of biomolecular condensates. The findings are **convincing**, supported by a clear physical model and systematic simulations showing effects on phase behavior, packing, and dynamics. Some clarification and broader context would improve the article, but it provides a foundation that will be of use for developing more realistic coarse-grained interaction schemes.

# Referee report: Reviewer #1 (Public review)

**DOI:** 10.7554/eLife.111124.3.sa1

This manuscript is very interesting and timely. By introducing the critical effects of desolvation barriers and solvent (water)-separated minima into the implicit-solvent potentials (of mean force, PMFs) for coarse-grained molecular dynamics simulations of biomolecular liquid-liquid phase separation (LLPS), this work fills a gap that should be apparent to researchers of protein folding in the past couple of decades but has so far escaped deserved attention such that these basic features of aqueous solvation have seldom, though not never, been invoked in recent studies of biomolecular condensates. Although the present paper deals almost exclusively with homopolymers, this work can be a foundation for the future development of a new, more physical coarse-grained interaction schemes for simulating amino acid sequence-dependent effects, which I presume is the authors' ongoing or next endeavor. The results presented in this manuscript are highly valuable.

However, there is room for improvement in the authors' description of (i) the broader impact of effects of desolvation barrier and solvent-separated minimum in the thermodynamics of biomolecular condensates, especially with regard to the ramifications on hydrostatic pressure-dependent effects; (ii) the physical implication of using a 20-parameter hydropathy scale rather than a 210-parameter pairwise amino acid interaction scheme; and (iii) temperature-dependent effects, including the authors' discussion of "enthalpic" and "entropic" contributions. In all these aspects, the authors' discussion should be put in a more comprehensive context of the existing literature. At a few other places, description of the methods and results should be clarified as well.

Comments on revised version

The authors have thoroughly and adequately addressed all my previous concerns and suggestions. The manuscript is now significantly improved in terms of clarity and proper placement in the context of prior works on desolvation effects in protein conformations.

# Referee report: Reviewer #2 (Public review)

**DOI:** 10.7554/eLife.111124.3.sa2

Summary:

This manuscript addresses an important and timely question in the molecular simulation of biomolecular condensates. Most residue-level coarse-grained models used for IDP phase separation employ implicit solvent and represent effective interactions through relatively simple pairwise potentials. While these models have been very useful, they usually do not explicitly distinguish direct contacts from solvent-separated interactions, nor do they include an energetic barrier associated with water removal. This manuscript attempts to address that limitation by introducing desolvation-inspired terms into coarse-grained models and examining their consequences for phase behavior, chain conformations, dense-phase packing, and dynamics.

The central idea is physically well motivated. Using a simple homopolymer model, the authors show that increasing the desolvation barrier suppresses phase separation, whereas stabilizing solvent-separated contacts enhances phase separation. They further show that solvent-separated interactions can reduce dense-phase over-compaction, which is a meaningful result given the known challenges in obtaining both accurate single-chain dimensions and realistic dense-phase properties from the same coarse-grained model. The finding that desolvation-like terms can reshape dense-phase packing without simply rescaling the overall interaction strength is interesting and could be useful for future model development. I also found the attempt to connect conformational changes across dilute and dense phases with thermal distance from the critical point to be intriguing. The dynamic analysis, including the FRAP-like simulations and the discussion of kinetic arrest during coarsening, adds another useful dimension to the work.

Overall, I think this is a useful and potentially important contribution.

Comments on revised version.

The authors have addressed my earlier comment regarding conformational changes between the dilute and condensed phases. One small additional suggestion is that they may find two related studies useful in this context: Devarajan et al., Nature Communications (2024), on relationships between dilute-phase conformations and condensate material properties, and Wang et al., Chemical Science (2024), which examines sequence-dependent conformational changes during condensation for both model polyampholyte sequences and naturally occurring IDPs. These studies may provide some complementary context for the discussion. This is simply a literature suggestion and does not affect my overall assessment of the revised manuscript.

# Author comment: Author response

**DOI:** 10.7554/eLife.111124.3.sa3

**Authors:** Kai Zhang, Zhiyu Peng, Wenfei Li, Wei Wang

**Affiliations:** Nanjing University Nanjing China; Nanjing University Nanjing China; Nanjing University Nanjing China; Nanjing University Nanjing China

The following is the authors’ response to the original reviews.

**Public Reviews:**

**Reviewer #1 (Public review):**

This manuscript is very interesting and timely. By introducing the critical effects of desolvation barriers and solvent (water)-separated minima into the implicit-solvent potentials (of mean force, PMFs) for coarse-grained molecular dynamics simulations of biomolecular liquid-liquid phase separation (LLPS), this work fills a gap that should be apparent to researchers of protein folding in the past couple of decades but has so far escaped deserved attention such that these basic features of aqueous solvation have seldom, though not never, been invoked in recent studies of biomolecular condensates. Although the present paper deals almost exclusively with homopolymers, this work can be a foundation for the future development of a new, more physical coarse-grained interaction scheme for simulating amino acid sequence-dependent effects, which I presume is the authors' ongoing or next endeavor. The results presented in this manuscript are highly valuable.

We thank the reviewer for all these positive comments.

However, there is room for improvement in the authors' description of (i) the broader impact of effects of desolvation barrier and solvent-separated minimum in the thermodynamics of biomolecular condensates, especially with regard to the ramifications on hydrostatic pressure-dependent effects; (ii) the physical implication of using a 20-parameter hydropathy scale rather than a 210-parameter pairwise amino acid interaction scheme; and (iii) temperature-dependent effects, including the authors' discussion of "enthalpic" and "entropic" contributions. In all these aspects, the authors' discussion should be put in a more comprehensive context of the existing literature. At a few other places, the description of the methods and results should be clarified as well. Accordingly, the authors should revise the manuscript to address the following items thoroughly within the revised manuscript (not merely in the response letter) with the additional references mentioned below included in the revised discussion:

(1) In several places, e.g., on line 77 (p.2), the authors appear to suggest that "implicit-solvent representation" is the origin of the deficiency in commonly utilized coarse-grained potentials that this study is aiming to rectify. But desolvation barriers and solvent-separated minima are also features of implicit-solvent representations; they are just features that should be incorporated in more accurate implicit-solvent potentials. This point is stated quite clearly and accurately in the Abstract (p.1) but not consistently in the rest of the text. The authors should check the entire text carefully to ensure that a coherent, accurate perspective is presented.

We thank the reviewer for pointing out this important issue. We agree that implicit-solvent representation itself is not the origin of the deficiency. Our intention is to incorporate desolvation-inspired effective terms within an implicit-solvent coarse-grained framework, because many commonly used implicit-solvent potentials do not directly account for the desolvation features, such as the desolvation barrier and the solvent-separated potential well.

We have revised the Abstract, Introduction, Results, and Discussion to make this distinction consistent throughout the manuscript. The revised text now emphasizes that the model remains an implicit-solvent CG model, but contains additional effective terms inspired by desolvation features observed in all-atom PMFs.

Corresponding changes:

(1) (page 1, lines 17–20) The Abstract identifies the model as an implicit-solvent CG model with added desolvation terms:

"Here, guided by all-atom simulations and experimental measurements, we develop a desolvation-aware implicit-solvent CG model by incorporating residue-level desolvation terms directly into the pairwise energy function and apply it to investigate LLPS of intrinsically disordered proteins."

(2) (page 2, lines 80–81) The Introduction retains the implicit-solvent description of existing residue-level CG models:

"Despite these advances, most residue-level CG models rely on implicit solvent representations, in which individual water molecules are not explicitly represented."

(3) (page 2, lines 87–89) The specific limitation is identified as the absence of a direct account of the multi-step desolvation process:

"More importantly, conventional implicit-solvent CG models used for LLPS usually do not directly account for the multi-step desolvation process that accompanies the transition from a dilute solution to a dense condensate."

(4) (page 4, lines 172–174) The added terms are described as part of a desolvation-inspired effective potential:

"Together, these parameters shape the desolvation-inspired effective potential and modulate the statistical balance between direct-contact and solvent-separated configurations."

(5) (page 15, lines 521–522) The Discussion restates the implicit-solvent model framework:

"To address this challenge, we developed a desolvation-aware implicit-solvent CG framework that incorporates desolvation barrier and solvent-separated terms into the pairwise potential."

(2) In the discussion of the importance of desolvation barriers and solvent-separated minima in the Introduction (pp.1-3), connections should be drawn to recent works that utilize these PMF features to rationalize hydrostatic pressure (P)-modulated effects on biomolecular LLPS, including the P-dependent reentrant phase separation of alpha elastin; see Cinar et al. (2019) Chem Eur J 25:13049 (https://chemistryeurope.onlinelibrary.wiley.com/doi/full/10.1002/chem.201902210) and references therein, especially discussions around Figures 10, 11 & 13 in this reference.

We thank the reviewer for bringing this literature to our attention. We agree that pressure-modulated LLPS provides important context for the physical relevance of desolvation barriers and solvent-separated minima. We have therefore expanded the Introduction and Discussion to connect our model to prior work on hydrostaticpressure-dependent condensate behavior, including pressure-dependent reentrant phase separation of alpha-elastin.

Corresponding changes:

(1) (page 3, lines 93–95) The Introduction connects the PMF features to hydrostaticpressure-dependent LLPS:

"Related studies on hydrostatic pressure effects have further suggested that desolvation barriers and solvent-separated minima can help rationalise pressure-modulated LLPS behaviors, including the pressure-dependent reentrant phase separation of α-elastin Cinar et al. (2019, 2018)."

(2) (page 15, lines 533–537) The Discussion states the pressure-dependent implication conservatively:

"These findings may also provide a useful physical basis for future studies of pressure-dependent condensate behavior, as pressure-induced changes in hydration, solvent-separated states, and desolvation barriers have been proposed to contribute to pressure-modulated and reentrant LLPS Dias and Chan, 2014; Cinar et al. (2019, 2018)."

(3) In the lower panels of Figures 2D, E (p.5), what do the differently colored small circles in the double-minimum free energy profiles represent? Does the color shading have the same meaning as that in the upper panels? If so, what do the positions of the circles on the free energy profile represent? The authors should clarify this.

We thank the reviewer for identifying this ambiguity. The small circles in the lower panels of Figures 2D and 2E are qualitative schematic representations of residue-pair configurations along the effective pair-potential profile. Their blue and green colors distinguish the barrier-variation and solvent-separated-well cases, respectively; they are not a quantitative scale and do not encode temperature or population magnitude. The positions of the circles indicate the direct-contact, barrierregion, or solvent-separated regions, while the density of circles schematically represents the population of configurations.

We have clarified this interpretation in the Figure 2 caption and aligned the Results text with the redistribution among direct-contact, barrier-region, and solvent-separated states.

Corresponding changes:

(1) (page 6, Figure 2D and E lower panels) The schematics distinguish low and high ε_b or ε_ss and use the density and position of the circles to depict populations in the direct-contact, barrier-region, and solvent-separated regions; the blue and green colors distinguish the two parameter families and are not a quantitative scale.

(2) (page 6, Figure 2 caption) The caption defines the population encoding used in the lower panels:

"The lower panels schematically illustrate how changes in ε_b_ and ε_ss_ alter the distribution of residue-pair configurations. The small circles indicate schematic populations of residue-pair configurations along the potential profile, with denser circles representing a higher population."

(3) (page 5, lines 210–213) The Results text connects the schematics to redistribution among the three residue-pair states:

"These opposing effects suggest that the desolvation potential regulates macroscopic phase behavior by redistributing residue-pair configurations between direct-contact, barrier-region, and solvent-separated states (lower panels of Figure 2D and E)."

(4) The discussion regarding entropy and enthalpy around Figure 2 is quite confusing as it stands. What do the authors mean exactly by the association of entropy or enthalpy with the desolvation barrier of the solvent-separated minimum? Are they referring to conformational entropy?

We thank the reviewer for pointing out this ambiguity. We agree that our original wording around entropy and enthalpy could be misleading, because it might imply a rigorous thermodynamic decomposition of the PMF. In the revised manuscript, we have therefore clarified that the effect of the desolvation barrier refers to an entropyrelated configurational restriction of residue-pair configurations near the barrier region, rather than the overall conformational entropy of the entire chain. We also replaced the previous "enthalpic stabilization" wording with "effective free-energy stabilization" to avoid implying that the solvent-separated minimum is treated as a purely enthalpic contribution.

Corresponding changes:

(1) (page 5, lines 199–201) The barrier effect is described in terms of the sampled residue-pair population:

"Analysis of residue-residue radial distribution functions showed that higher ε_b_ suppresses the population of configurations near the barrier region (Figure 2—figure Supplement 1C)."

(2) (page 5, lines 201–202) The entropy-related statement is restricted to configurational sampling near the barrier:

"This reduction in the statistical weight of barrier-region configurations can be interpreted as an entropy-related configurational restriction and thus disfavors phase separation."

(3) (page 5, lines 209–210) The solvent-separated minimum is described as an effective free-energy contribution:

"This solvent-separated minimum provides effective free-energy stabilization for water-mediated configurations and thereby promotes phase separation."

(4) (page 6, Figure 2D and E lower panels) The schematic headings are "Barrier-mediated Restriction" and "Solvent-separated Stabilization", avoiding a strict entropy-enthalpy decomposition.

(5) Do the authors assume that the PMF (effective implicit-solvent potential) is a purely enthalpic term? It appears to be the authors' assumption. If so, the assumption has to be stated clearly in their discussion of "entropy" vs "enthalpy" around Figure 2.

We thank the reviewer for raising this important point. We do not assume that the PMF obtained from all-atom simulations is a purely enthalpic term. The PMF is a free-energy profile that contains enthalpic and entropic contributions. The current manuscript defines the PMF as −*k*_B_ *T* ln*P*(r), uses the all-atom PMFs to motivate a nonbonded effective coarse-grained potential, and describes the solvent-separated minimum as providing effective free-energy stabilization. We do not perform a rigorous enthalpy-entropy decomposition, and the revised wording avoids implying such a decomposition.

Corresponding changes:

(1) (page 16, lines 594–595) The Methods define the PMF as a free-energy profile obtained from the radial probability density:

"The potential of mean force (PMF) was computed as PMF(r) = −k_B_T ln P(r), where P(r) is the radial probability density obtained from the production trajectory."

(2) (page 5, Figure 1 caption) The CG interaction is labeled as an effective potential rather than as an enthalpic PMF decomposition:

"Pairwise effective potential incorporating desolvation-inspired terms. Different curves correspond to different desolvation parameters."

(3) (page 4, lines 172–174) The parameters are described as shaping an effective potential:

"Together, these parameters shape the desolvation-inspired effective potential and modulate the statistical balance between direct-contact and solvent-separated configurations."

(4) (page 5, lines 209–210) The solvent-separated contribution is described using free-energy language:

"This solvent-separated minimum provides effective free-energy stabilization for water-mediated configurations and thereby promotes phase separation."

(6) Closely related to points 3-5 above, it should be stated clearly that the "temperature" used in the authors' simulations does not represent experimental temperature if the authors are using purely enthalpic effective potentials because PMFs are in fact temperature-dependent. This clarification is necessary to avoid misunderstanding. In this regard, it should be noted that temperature-dependent effective interactions have been used for modeling biomolecular condensates in analytical theory (Lin, Song, Forman-Kay & Chan, J Mol Liq 2017, already in the citation list) as well as in coarse-grained molecular dynamics simulations [Dignon et al. (2019) ACS Cent Sci 5:821-830 (https://pubs.acs.org/doi/10.1021/acscentsci.9b00102); Chakravarti & Joseph (2025) Protein Sci 34:e70284 (https://onlinelibrary.wiley.com/doi/10.1002/pro.70284)]. The latter two studies, not cited currently, are particularly relevant and thus should be cited because the authors may wish to incorporate temperature-dependent features in their ongoing or future effort in constructing a more comprehensive coarse-grained interaction scheme for biomolecular LLPS simulation.

We agree with the reviewer that the simulation temperature should be interpreted carefully. In the present simulations, the effective potential is temperature-independent within each chosen parameter set. Therefore, the reduced temperature primarily serves as a model temperature controlling the relative strength of thermal fluctuations, rather than as a direct experimental temperature. We have clarified this point in the revised manuscript and added relevant references on temperature-dependent effective interactions, which represent an important direction for future model development.

Corresponding changes:

(1) (page 4, lines 177–179) The manuscript states that the absolute simulation temperature is not an experimental temperature:

"Because the effective interaction parameters used in the model are temperature-independent, the absolute simulation temperature should not be directly interpreted as an experimental temperature."

(2) (page 4, lines 182–184) The reduced temperature is identified as a model temperature:

"Accordingly, T^*^ should be interpreted primarily as a model temperature that controls the relative strength of thermal fluctuations, rather than as having a direct quantitative correspondence with experimental temperature."

(3) (page 14, lines 481–485) The FUS LC temperature comparison is framed cautiously:

"It is worth noting that the residue-level coarse-grained models used here employ temperature-independent effective interaction parameters. As a result, the temperature values reported here cannot be interpreted as quantitatively equivalent to experimental temperatures, particularly when they deviate substantially from room-temperature conditions."

(4) (page 15, lines 563–567; continues on page 16, lines 568–569) The Discussion identifies temperature-dependent effective interactions and a corresponding future extension:

"In addition, effective interactions themselves can be temperature-dependent, as demonstrated in analytical theories and coarse-grained simulations of biomolecular condensates Lin et al. (2017); Dignon et al. (2019); Chakravarti and Joseph (2025). Future extensions of the model could therefore incorporate residue-specific and temperature-dependent desolvation parameters derived from bottom-up parameterization or expanded experimental datasets, thereby enhancing predictive accuracy for sequence-dependent LLPS."

(7) In tackling "entropy" vs "enthalpy", it should be noted that the temperature dependence of the effective interactions entails an entropic contribution (which is itself temperature dependent) in addition to conformational entropy. As for the effective potential with desolvation barrier and solvent-separated minimum, it should be noted that the decomposition into entropic and enthalpic contributions at the direct contact, desolvation barrier, and solvent-separated minimum can be dramatically different, see, e.g., MaCallum et al. (2007) PNAS 104:6206-6210 (https://www.pnas.org/doi/full/10.1073/pnas.0605859104) and references therein.

We thank the reviewer for this important clarification. We agree that temperature-dependent effective interactions can contain entropic contributions beyond conformational entropy and that the balance of enthalpic and entropic contributions may differ among the direct-contact minimum, desolvation barrier, and solvent-separated minimum. The present model does not decompose the PMF into temperature-dependent enthalpic and entropic components; accordingly, we have avoided assigning those components to individual PMF features. The Discussion cites explicit-solvent PMF analyses when noting residue-pair and temperature dependence and identifies temperature-dependent desolvation parameters as an important future extension.

Corresponding changes:

(1) (page 15, lines 561–563) The Discussion cites explicit-solvent PMF work when noting residue-pair and temperature dependence:

"Explicit-solvent PMF analyses have shown that desolvation barrier heights and solvent-separated minima can differ substantially among residue pairs and may also exhibit temperature dependence Cinar et al. (2019); Debiec et al. (2014); MacCallum et al. (2007)."

(2) (page 15, lines 563–566) The manuscript states that effective interactions can themselves depend on temperature:

"In addition, effective interactions themselves can be temperature dependent, as demonstrated in analytical theories and coarse-grained simulations of biomolecular condensates Lin et al. (2017); Dignon et al. (2019); Chakravarti and Joseph (2025)."

(3) (page 15, lines 566–567; continues on page 16, lines 568–569) Temperature-dependent desolvation parameters are identified as a future model extension:

"Future extensions of the model could therefore incorporate residue-specific and temperature-dependent desolvation parameters derived from bottom-up parameterization or expanded experimental datasets, thereby enhancing predictive accuracy for sequence-dependent LLPS."

(8) P.7, line 340: The proportionality relation follows directly from the standard FloryHuggins result T_c = T chi(T)/chi_c, thus the proportionality constant is exactly 1/chi_c. Is this the standard relation that the authors are invoking here? The authors should clarify this.

We thank the reviewer for pointing out the missing intermediate steps. Yes, the relation we invoked is based on the standard Flory-Huggins critical condition. In the revised manuscript, we have expanded the derivation to explicitly show how the critical condition chi(T_c) = chi_c leads to the relation between chi(T_sim) - chi_c and the normalized thermal distance (T_c - T_sim)/T_sim.

We also revised the wording to avoid presenting this as a universal law. The relation is now presented as a simulation-supported trend within the present model, rationalized by a simplified linear-response assumption between Delta R_g and the excess interaction strength.

Corresponding changes:

(1) (page 7, lines 263–264) The critical-condition substitution is now shown explicitly:

"At the critical point, χ(T_c_) = χ_c_, which gives ε_eff_ = k_B_T_c_χ_c_. Substituting this relation into the expression for χ(T_sim_) yields χ(T_sim_) = χ_c_T_c_/T_sim_."

(2) (page 7, line 265) The resulting relation is written as Equation (2):

"χ(T_sim_) − χ_c_ = χ_c_ (T_c_ − T_sim_)/T_sim_."

(3) (page 7, lines 266–267) The fixed-chain-length assumption is stated explicitly:

"For systems with the same chain length, χ_c_ is a fixed constant. Thus, the deviation from the critical interaction parameter is directly related to the rescaled thermal distance (T_c_ − T_sim_)/T_sim_."

(9) The study on dynamic consequences on pp.8-11 is interesting, but clarifications are necessary:

(i) The vertical schematic in Figure 4A should be explained in detail in its entirety. As it stands, no explanation is provided either in the figure caption or in the text. In particular, what does "elasticity driven" refer to?

(ii) The top snapshot in Figure 4A is labeled t_sim = 0 ns. Does it mean that the snapshot shown is the only chain configuration that the authors used to start the simulation, and that the snapshot does NOT represent the result of any time evolution, no matter how short the duration is? However, if that is the case, why is this snapshot identified with spinodal decomposition if it is not the product of a time evolution from a more homogeneous configuration?

(iii) Related to (ii) - do the rectangular boxes shown represent the entire simulation box or just part of the box containing the polymer chains? One would imagine that if the top snapshot represents spinodal decomposition, the simulation would have been started at a more uniform distribution a short time prior? Why is this not the case?

(iv) What precisely do the small yellow beads and black-colored springs in the zoomin image of Figure 4E represent?

We thank the reviewer for all these inspiring comments and questions. We agree that the original Figure 4 schematic did not sufficiently explain the sequence of dynamical events and the meaning of several graphical elements. We have therefore revised both the Figure 4 caption and the Results text to make the schematic self-contained and to clarify how it relates to the quantitative analyses in Figure 4F and G.

First, we replaced the phrase "elasticity driven" with a more precise description of "viscoelastic resistance". In the revised text, interfacial tension is described as favoring domain fusion thermodynamically, whereas transient inter-chain network connectivity within dense domains generates viscoelastic resistance to the deformation required for coalescence. This revision avoids implying that elasticity is the driving force and instead identifies it as a resistance that delays domain fusion kinetically during the plateau regime.

Second, we clarified the meaning of t_sim = 0 ns and its relation to spinodal decomposition. The system was equilibrated at a supercritical temperature to obtain a homogeneous one-phase state and was then instantaneously quenched to the target temperature. The label t_sim = 0 ns denotes the first snapshot immediately after the quench. It is not the only initial configuration used in all simulations; the reported kinetic metrics were averaged over six independent slab simulation replicas.

The t_sim = 0 ns snapshot is therefore described as a homogeneous but thermodynamically unstable post-quench state. Spinodal decomposition refers to the subsequent amplification of the initial density fluctuations after the quench, including the development of interconnected density fluctuations within 1-2 ns, rather than to a preceding evolution represented by the t_sim = 0 ns snapshot.

Third, we clarified that the rectangular snapshots in Figure 4A show the entire simulation box viewed along the z-axis. The subsequent snapshots show how post-quench density fluctuations grow and reorganize into dense domains during spinodal decomposition.

Finally, we clarified the symbols in the zoom-in schematic of Figure 4E. Yellow beads now denote residues involved in transient inter-chain contacts, and black springs denote schematic network connections formed by these contacts. These elements are meant to illustrate transient network connectivity and are not additional simulated particles or force-field terms.

Corresponding changes:

(1) (page 10, Figure 4A) The vertical schematic now labels the progression as "Thermodynamic instability", "Kinetic arrest (viscoelastic resistance)", "Domain coarsening (interfacial-tension dominated)", and "Dynamic equilibrium (chain self-diffusion)".

(2) (page 10, Figure 4E) The plateau schematic labels the competing effects as "Interfacial Tension" and "Transient network resistance".

(3) (page 10, Figure 4 caption) The caption defines the snapshots and the vertical schematic:

"Upper snapshots show the simulation box along the z-axis at t_sim_ = 0, 10, and 500 ns. The vertical schematic summarizes the dynamical progression described in the main text, from the post-quench spinodal instability to kinetic arrest, domain coarsening, and dynamic equilibrium."

(4) (page 11, lines 368–370) The first recorded time point after the quench is defined explicitly:

"Here, t_sim_ = 0 ns denotes the first snapshot immediately after the temperature quench, corresponding to a homogeneous but thermodynamically unstable nonequilibrium state."

(5) (page 10, Figure 4 caption) The yellow beads and black springs are defined:

"In the zoom-in view, yellow beads denote residues involved in transient inter-chain contacts, and black springs denote schematic network connections formed by these contacts."

(6) (page 12, lines 401–404) The Results explain the physical meaning of the transient network:

"In the zoom-in schematic in Figure 4E, this transient network is represented by connections between residues involved in inter-chain contacts, illustrating how multivalent interactions can resist domain deformation during the plateau regime."

(10) In discussing dynamic effects, it is useful to draw connections to related works on the effect of chain flexibility on "aging" of condensate [Biswas & Potoyan (2024) PRX 45:9222-9245 (https://journals.aps.org/prxlife/abstract/10.1103/PRXLife.2.023011)] and characterization of viscoelasticity in simulations of biomolecular condensates [Tejedor et al. (2023) J Phys Chem B 127:4441-4459 (https://pubs.acs.org/doi/10.1021/acs.jpcb.3c01292)], as the effects of desolvation can be explored further based on these prior works.

We thank the reviewer for these important references. We have added connections to simulation studies of condensate viscoelasticity and aging. The revised manuscript now places our dynamic results in the context of transient network connectivity, chain flexibility, sticker lifetime, desolvation-associated rigidification, and viscoelastic or aging-like material behavior.

We present these connections conservatively as relevant context and as future directions for extending the current model, rather than claiming a new universal dynamic mechanism.

Corresponding changes:

(1) (page 12, lines 397–399) The dynamics section cites simulation-based rheological analyses of condensate viscoelasticity:

"Similar viscoelastic effects have recently been quantified in molecular simulations of biomolecular condensates using rheological analyses of time-dependent material properties Tejedor et al. (2023)."

(2) (page 12, lines 399–401) The manuscript connects condensate aging to chain flexibility, sticker lifetime, and desolvation-associated rigidification:

"Molecular simulations of condensate aging have further highlighted the roles of chain flexibility, sticker lifetime, and desolvation-associated rigidification in promoting more solid-like states Biswas and Potoyan (2024)."

(3) (page 12, lines 423–427) The kinetic interpretation is connected to viscoelastic andaging-like behavior:

"The sensitivity of kinetic arrest and coarsening dynamics to desolvation parameters underscores the importance of incorporating desolvation features into coarse-grained potentials for more physically plausible molecular simulations of LLPS, especially when connecting microscopic interaction lifetimes to emergent viscoelastic or ageing-like material behavior."

(4) (page 16, lines 569–572) The Discussion identifies simulation-based rheological analysis as a future direction:

"An additional direction would be to combine these potentials with simulation-based rheological analyses to quantify how desolvation reshapes condensate viscoelasticity, aging-like maturation, and long-time material relaxation Tejedor et al. (2023); Biswas and Potoyan (2024)."

(11) Much of the present study is based on the original HPS formulation of Dignon et al. (2018). In this regard and also in anticipation of future development of improved interaction schemes, several issues should be stated and discussed, even if briefly:

(i) The original HPS model has a basic shortcoming in accounting for the relative interaction strengths of, among others, arginine vs lysine residues [Das et al. (2020) PNAS 117:28795-28805 (https://www.pnas.org/doi/10.1073/pnas.2008122117)].

(ii) Compared to 210-parameter pairwise interaction schemes, such as KH in Dignon et al. (2018) and Joseph et al. (2021), the 20-parameter interaction scheme is likely too restrictive to account for pairwise amino acid residue interactions [Wessén et al. (2022) J Phys Chem B 45:9222-9245 (https://pubs.acs.org/doi/10.1021/acs.jpcb.2c06181)].

(iii) The height of the desolvation barrier may vary significantly for different amino acid residue pairs, see, e.g., Figure 11 of Cinar et al. (2019) mentioned above (and references therein). The authors should discuss these nuances in the revised version. They may also wish to take them into consideration in future investigations.

We thank the reviewer for the suggestion to clarify these limitations. We have revised the Discussion to acknowledge explicitly the limitations of the 20-parameter hydropathy-scale representation relative to more flexible 210-parameter pairwise interaction schemes for describing amino-acid-pair interactions. We have also added discussion emphasizing that future desolvation-aware models should incorporate residue-pair-specific parameters for the desolvation barrier and solvent-separated potential well.

Corresponding changes:

(1) (page 13, lines 445–446) The scope of the averaged baseline parameterization is stated explicitly:

"This uniform parameterization captures the generic desolvation features of the PMFs but does not resolve residue-pair-specific variations in desolvation energetics."

(2) (page 15, lines 551–554) The Discussion identifies the limitation of the 20-parameter HPS representation, including Arg/Lys interactions:

"In particular, HPS-type models use a 20-parameter hydropathy-scale representation, which is useful for capturing generic IDP phase behavior but is not flexible enough to resolve residue-pair-specific chemical effects, such as the distinct interaction patterns of arginine and lysine residues Das et al. (2020)."

(3) (page 15, lines 554–557) The greater flexibility of 210-parameter pairwise schemes is described:

"More general 210-parameter pairwise interaction schemes, such as KH-type and related residue-pair-specific models, provide greater flexibility for encoding amino acid-pair preferences and capturing sequence-specific interaction heterogeneity Dignon et al. (2018b); Joseph et al. (2021); Wessén et al. (2022)."

(4) (page 15, lines 557–561) The limitation of using one averaged desolvation parameter set is stated:

"Second, the present desolvation model employs a single set of averaged parameters (α_b_, α_ss_) for all residue pairs. While this simplification is effective for isolating the generic physical consequences of desolvation, it has limitations in describing the pair-specific variations in the desolvation barrier and the solvent-separated minimum."

(5) (page 15, lines 566–567; continues on page 16, lines 568–569) Residue-specific and temperature-dependent parameters are identified as a future extension:

"Future extensions of the model could therefore incorporate residue-specific and temperature-dependent desolvation parameters derived from bottom-up parameterization or expanded experimental datasets, thereby enhancing predictive accuracy for sequence-dependent LLPS."

**Reviewer #2 (Public review):**

Summary:

This manuscript addresses an important and timely question in the molecular simulation of biomolecular condensates. Most residue-level coarse-grained models used for IDP phase separation employ implicit solvent and represent effective interactions through relatively simple pairwise potentials. While these models have been very useful, they usually do not explicitly distinguish direct contacts from solvent-separated interactions, nor do they include an energetic barrier associated with water removal. This manuscript attempts to address that limitation by introducing desolvation-inspired terms into coarse-grained models and examining their consequences for phase behavior, chain conformations, dense-phase packing, and dynamics. Strengths:

The central idea is physically well motivated. Using a simple homopolymer model, the authors show that increasing the desolvation barrier suppresses phase separation, whereas stabilizing solvent-separated contacts enhances phase separation. They further show that solvent-separated interactions can reduce densephase over-compaction, which is a meaningful result given the known challenges in obtaining both accurate single-chain dimensions and realistic dense-phase properties from the same coarse-grained model. The finding that desolvation-like terms can reshape dense-phase packing without simply rescaling the overall interaction strength is interesting and could be useful for future model development. I also found the attempt to connect conformational changes across dilute and dense phases with thermal distance from the critical point to be intriguing. The dynamic analysis, including the FRAP-like simulations and the discussion of kinetic arrest during coarsening, adds another useful dimension to the work.

Weaknesses:

At the same time, there are several places where the manuscript would benefit from more careful framing. First, the desolvation terms are still effective coarse-grained parameters rather than a direct representation of water molecules. The language sometimes gives the impression that desolvation is being treated explicitly, whereas the model introduces desolvation-inspired effective interactions into an implicitsolvent framework.

We thank the reviewer for the positive assessment and constructive suggestions. We agree that the desolvation terms should be described as effective coarse-grained parameters rather than explicit water molecules. We have revised the manuscript to describe the model as a desolvation-aware implicit-solvent coarse-grained framework with desolvation-inspired effective interaction terms.

Corresponding changes:

(1) (page 1, lines 17–20) The Abstract identifies the model as an implicit-solvent CG model:

"Here, guided by all-atom simulations and experimental measurements, we develop a desolvation-aware implicit-solvent CG model by incorporating residue-level desolvation terms directly into the pairwise energy function and apply it to investigate LLPS of intrinsically disordered proteins."

(2) (page 4, lines 151–153) The Results describe the added contributions as desolvation-related effective terms:

"These observations underscore the importance of incorporating desolvation-related effective terms and exploring the effects of different desolvation strengths on the thermodynamics and kinetics of protein LLPS."

(3) (page 4, lines 172–174) The pair interaction is described as a desolvation-inspired effective potential:

"Together, these parameters shape the desolvation-inspired effective potential and modulate the statistical balance between direct-contact and solvent-separated configurations."

(4) (page 15, lines 541–543) The Discussion emphasizes that the framework retains water-mediated features within an implicit-solvent representation:

"By retaining key water-mediated features while preserving the computational efficiency of implicit-solvent representations, this framework provides a mechanistic means to decouple overall phase-separation propensity from condensed-phase packing."

Second, the conformational analysis is interesting, but the broader context of prior work on dilute-to-dense phase conformational reorganization of IDPs could be more clearly discussed. This would help clarify what is new in the present work, whether it is the conformational change itself, its dependence on desolvation terms, or the proposed scaling with distance from the critical point.

We thank the reviewer for this suggestion. We agree that the conformational change itself should be placed in the context of prior work. The contribution of the present analysis is not simply the observation that IDP conformations can reorganize upon condensation. Rather, we examine how desolvation-inspired effective terms modulate dilute- and dense-phase conformations and how the conformational change correlates with thermal distance from the critical point within the present model.

We have revised the Results section discussing Figure 3 to cite prior work and to state the interpretation of ΔR_g more clearly.

Corresponding changes:

(1) (page 6, lines 230–232) Prior work on conformational reorganization upon condensation is cited:

"Previous studies have shown that IDP condensation can reorganize chain conformations by redistributing the balance between intra-chain and inter-chain interactions Wei et al. (2017); Hazra and Levy (2021); Tesei et al. (2021); von Bülow et al. (2025)."

(2) (page 7, lines 241–242) The phase-dependent conformational response to the desolvation parameters is introduced:

"In addition to the difference between dilute- and dense-phase conformations, varying the desolvation parameters further reveals a phase-dependent conformational response (Figure 3A–C)."

(3) (page 7, lines 243–244) The stronger response in the dilute phase is stated directly:

"Increasing ε_b_ or decreasing ε_ss_ shifts the dilute-phase R_g_ distributions toward larger values, whereas the dense-phase R_g_ remains comparatively insensitive to these parameter changes."

(4) (page 7, lines 247–249) The source of the desolvation-dependent variation in ΔR_g is identified:

"As a result, the desolvation-dependent variation in ΔR_g_ = R_g_^dense^ − R_g_^dilute^ arises predominantly from the conformational changes of isolated chains in the dilute phase."

(5) (page 7, lines 254–256) The observed relationship is presented as an approximate trend in the simulated systems:

"Notably, data from the simulated systems approximately follow a common trend, revealing a strong correlation between the magnitude of conformational change and the thermal distance to the phase transition point (R^2^ = 0.942, Figure 3D)."

Third, the dynamic results are potentially useful, but the manuscript should more clearly articulate what is nontrivial beyond the expected slowing of local rearrangements by an added barrier in the potential.

Overall, I think this is a useful and potentially important contribution.

We thank the reviewer for this constructive comment and the positive overall assessment. We have revised the dynamics section to clarify that the nontrivial result lies in the competition between two effects: although the desolvation barrier directly slows local rearrangements, its reduction of dense-phase packing can reverse the net mobility trend at fixed temperature. At matched thermodynamic quench depth, the intrinsic slowing associated with energy-landscape roughness becomes evident. We also clarified that desolvation modulates transient kinetic arrest and domain-scale coarsening, not only local rearrangements.

Corresponding changes:

(1) (page 11, lines 353–357) The fixed-temperature and matched-quench-depth analyses are summarized as opposing contributions:

"Together, the fixed-temperature and renormalized analyses in Figure 4C and D reveal two distinct and opposing contributions of desolvation to condensate dynamics. At fixed temperature, increasing ε_b_ loosens dense-phase packing and thereby increases the measured diffusion coefficient, whereas at matched thermodynamic quench depth, the same parameter change suppresses chain mobility by roughening the microscopic energy landscape and slowing local rearrangements."

(2) (page 11, lines 358–361) The multiscale interpretation is stated explicitly:

"Condensate dynamics therefore emerge from a balance between density-regulated mobility and energy-landscape-regulated mobility, with macroscopic packing determining the dominant trend and microscopic barrier roughness imposing an additional kinetic modulation. This interplay highlights how desolvation reshapes condensate dynamics across multiple physical scales."

(3) (page 12, lines 421–423) The dynamics section distinguishes the result from simple local slowing:

"This picture shows that desolvation does more than slow down local chain rearrangements through an added barrier. It also regulates the balance between fluctuation growth, transient arrest, and domain coarsening, thereby shaping the evolution of phase-separated domains."

**Reviewer #2 (Recommendations for the authors):**

(1) The model is physically motivated and useful, but I would encourage the authors to be more precise in describing the added terms as desolvation-inspired effective interactions rather than explicit desolvation.

We thank the reviewer for the comment and suggestion. We have revised the manuscript accordingly and describe the added terms as desolvation-inspired effective interactions within an implicit-solvent CG framework throughout the Abstract, Results, and Discussion. More detailed changes are provided in our response to the first point raised in Reviewer #2's Public Review.

(2) The desolvation barrier is introduced as part of the equilibrium pair potential, and therefore it is expected to affect not only kinetics but also the phase boundary through changes in the configurational partition function. The manuscript would benefit from clarifying this point, since the term "barrier" may otherwise suggest a primarily kinetic role. In particular, the authors should explain whether the observed shift in T_c reflects a change in the effective pair attraction, for example, through the integrated Boltzmann weight or second virial coefficient, rather than only an entropic penalty associated with restricted configurations.

We thank the reviewer for this important point. We agree that the desolvation barrier is part of the equilibrium pair potential and therefore affects the phase boundary through the Boltzmann-weighted sampling of residue-pair configurations, not only through kinetic slowing.

Following this recommendation, we added a bead-level second virial coefficient analysis based on the effective pair potential. This analysis provides a pair-potentiallevel measure of the integrated effective attraction and clarifies why increasing the barrier lowers T_c, whereas stabilizing the solvent-separated minimum raises T_c.

Corresponding changes:

(1) (page 5, lines 203–205) The equilibrium role of the barrier is stated explicitly:

"At the pair-potential level, the desolvation barrier modifies the equilibrium Boltzmann weight and thereby alters the integrated effective attraction, as quantified by the bead-level second virial coefficient B_2_."

(2) (page 5, lines 205–207) The barrier-dependent second virial coefficient is connected to the shift in critical temperature:

"Specifically, increasing ε_b_ makes B_2_/σ^3^ larger (Figure 2—figure Supplement 1G), indicating a weaker integrated effective attraction and providing a thermodynamic basis for the lower T_c_^*^."

(3) (page 5, lines 207–209) The solvent-separated-well trend is linked to a smaller second virial coefficient:

"By contrast, deepening the solvent-separated well ε_ss_ elevates T_c_^*^ (Figure 2E), which is associated with the enhanced population of solvent-separated configurations and a smaller B_2_/σ^3^ (Figure 2—figure Supplement 1D, H)."

(4) (page 18, lines 662–665) The Methods specify the integration range and the quantity used to compare integrated effective attraction:

"The upper limit of integration r_c_ is set as 3σ, which is sufficiently large to capture the full range of interactions while ensuring numerical convergence. The reduced value B_2_/σ^3^ was used to compare the integrated effective attraction under different desolvation parameters."

(5) (Figure 2—figure supplement 1G, H) The new panels report the integrated effective attraction:

"(G, H) Bead-level second virial coefficient (B_2_/σ^3^) calculated from the effective pair potential under varying ε_b_ at fixed ε_ss_ = 0.02 kcal/mol (G) and varying ε_ss_ at fixed ε_b_ = 3.12 cal/mol (H)."

(3) The conformational analysis in Figure 3 is interesting and potentially important. It would help to better place this result in the context of prior work showing dilute-todense phase conformational reorganization of IDPs, and to clarify what is new here beyond that broader observation.

We thank the reviewer for the comment and suggestion. We have revised the Results section discussing Figure 3 to place dilute-to-dense conformational reorganization of IDPs in the context of previous studies and then to emphasize the specific contribution of the present work.

The revised text clarifies that, within the present model, desolvation-inspired interactions mainly regulate chain conformations in the dilute phase, whereas dense-phase conformations remain comparatively insensitive. Detailed changes are provided in our response to the second point raised in Reviewer #2's Public Review.

(4) The proposed scaling between ΔR_g and distance from the critical point is intriguing, but the argument relies on simplifying assumptions. I would present this more as an empirical scaling supported by a plausible theoretical argument rather than a general result.

We thank the reviewer for this helpful suggestion. We agree that the correlation between Delta R_g and the distance from the critical point relies on simplifying assumptions and should not be presented as a general law. In the revised manuscript, we have softened the interpretation and now present this relationship as an empirical correlation supported by a simplified Flory-Huggins-based theoretical argument.

Corresponding changes:

(1) (page 7, lines 254–256) The relationship is described as an approximate trend in the simulated systems:

"Notably, data from the simulated systems approximately follow a common trend, revealing a strong correlation between the magnitude of conformational change and the thermal distance to the phase transition point (R^2^ = 0.942, Figure 3D)."

(2) (page 7, lines 256–258) The interpretation is limited to an association with thermal distance from the critical point:

"This result suggests that the conformational response to phase separation is closely associated with how far the system resides thermally from the critical point."

(3) (page 7, lines 263–264) The critical-condition derivation is shown explicitly:

"At the critical point, χ(T_c_) = χ_c_, which gives ε_eff_ = k_B_T_c_χ_c_. Substituting this relation into the expression for χ(T_sim_) yields χ(T_sim_) = χ_c_T_c_/T_sim_."

(4) (page 7, lines 268–272) The structural relation is explicitly introduced as a first-order linear-response approximation:

"The thermodynamic driving force χ(T_sim_) – χ_c_ can then be related to the structural observable ΔR_g_. Since ΔR_g_ captures the structural transition from an intrachain-interaction-dominated state in the dilute phase to an interchain-interaction-dominated state in the dense phase, we assume, as a first-order approximation, that this conformational shift responds approximately linearly to the excess interaction strength, expressed as ΔR_g_ ∝ [χ(T_sim_) − χ_c_]."

(5) (page 8, lines 285–287) The unscaled relation is labeled as an empirical scaling approximation:

"Although the complete relation in Equation (3) contains an additional T_sim_ factor, the unscaled quantities remain strongly correlated over the simulated range. We therefore use T_c_ − T_sim_ ∝ ΔR_g_ as an empirical scaling approximation."

(5) The dynamics section would benefit from a statement of what is nontrivial, since a desolvation barrier is expected to slow local rearrangements.

We thank the reviewer for the comment and suggestions. As described above, we have revised the dynamics section to clarify what is nontrivial beyond the expected slowing of local rearrangements by an added barrier. The revised text emphasizes that desolvation affects condensate dynamics through competing effects of macroscopic packing and microscopic energy-landscape roughness, and that it also regulates transient kinetic arrest and domain-scale coarsening. More detailed changes are provided in our response to the third point raised in Reviewer #2's Public Review.
